# An AMPK biosensor for *Caenorhabditis elegans*

**DOI:** 10.17912/micropub.biology.000596

**Published:** 2022-07-07

**Authors:** Lynly Carman, Ryan J Schuck, Edwin Li, Matthew D Nelson

**Affiliations:** 1 Department of Biology, Saint Joseph’s University, Philadelphia, PA

## Abstract

Adenosine monophosphate-activated kinase (AMPK) functions in a broad spectrum of cellular stress response pathways. Investigation of AMPK activity has been limited to whole-organism analyses in *Caenorhabditis elegans* which does not allow for observations of cellular heterogeneity, temporal dynamics, or correlation with physiological states in real time. We codon adapted the genetically-coded AMPK biosensor, called AMPKAR-EV, for use in *C. elegans* . We report heterogeneity of activation in different tissues (intestine, neurons, muscle) and test the biosensor in the context of two missense mutations affecting residues T243 and S244 on the AMPK α subunit, AAK-2, which are predicted regulatory sites.

**Figure 1. An AMPK biosensor for Caenorhabditis elegans:**
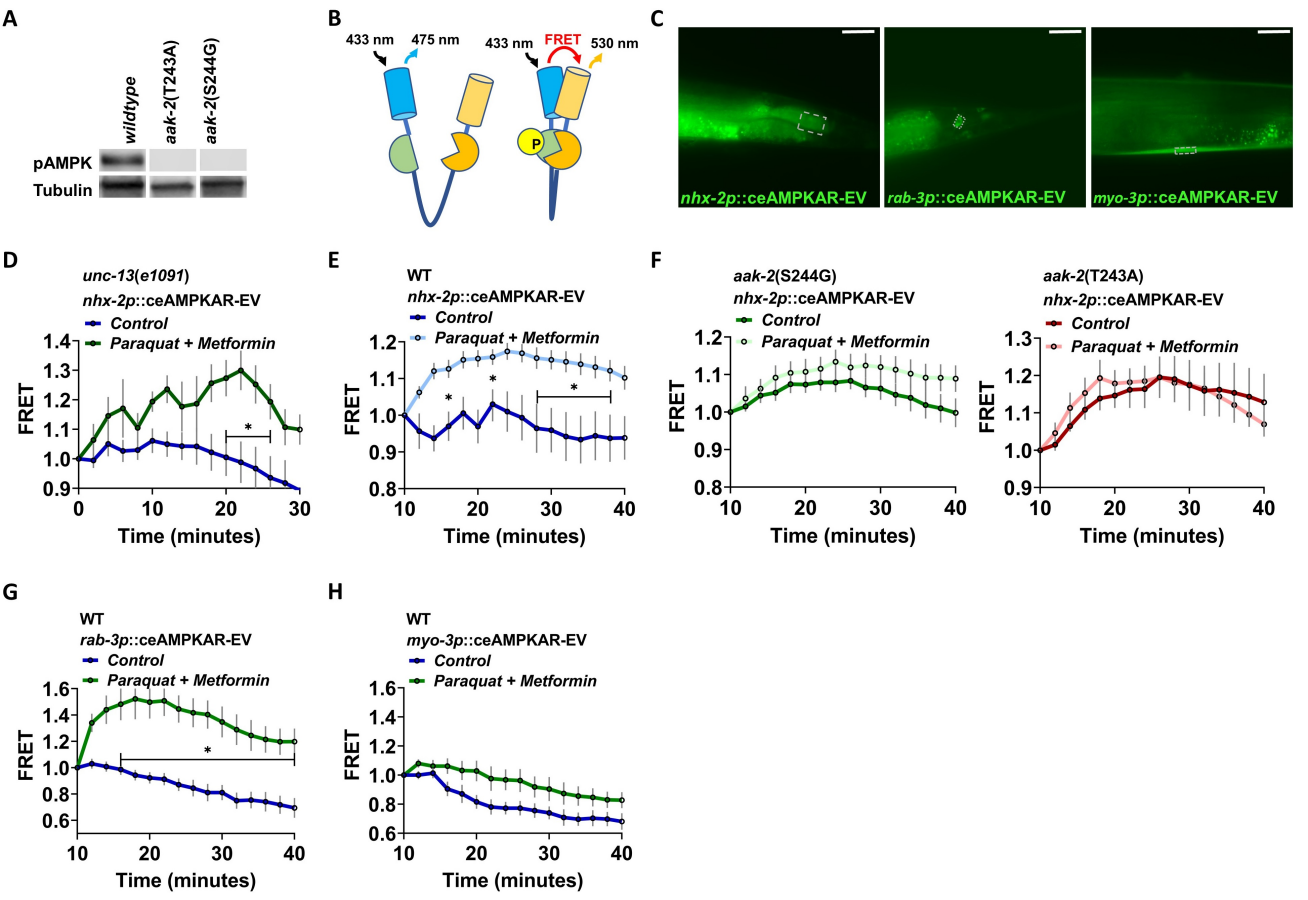
**A.** Phospho-AMPKα (Thr172) antibodies bind to wild-type AAK-2 but not AAK-2 from *aak-2* (T243A) and *aak-2* (S244G) animals. **B.** Schematic of the AMPKAR-EV biosensor structure and function. **C.** Representative image of *nhx-2p* ::ceAMPKAR-EV, *rab-3p* ::ceAMPKAR-EV, and *myo-3p* ::ceAMPKAR-EV transgenic animals. The defined ROI (box) shows where the FRET analyses were conducted, scale bar is 25µm. **D.** Paraquat and metformin exposure resulted in a significant increase in the normalized FRET ratio in the intestine over a period of 30 minutes (*p<0.05 at all points within the bracket; N=10). Statistical significance was calculated using two-way ANOVA followed by Sidak’s multiple comparisons test. **E.** Wild-type animals pre-treated with paraquat and metformin exhibit a significant increase in normalized FRET in their intestine over time (*p<0.05 including all points within the bracket; N=10). Statistical significance was calculated using two-way ANOVA followed by Sidak’s multiple comparisons test. **F.** *aak-2* (T243A) and *aak-2* (S244G) animals showed no difference in normalized FRET in their intestine over time between treated and control animals (N=10). Statistical significance was calculated using two-way ANOVA followed by Sidak’s multiple comparisons test. **G.** Wild-type animals that were pre-treated with paraquat and metformin exhibited a significant increase in normalized FRET in a tail neuron (DVA) over time (*p<0.05 at all points within the bracket; N=10). Statistical significance was calculated using two-way ANOVA followed by Sidak’s multiple comparisons test. **H.** Wild-type animals pre-treated with paraquat and metformin did not exhibit any changes in normalized FRET in a body wall muscle compared to controls (N=10). Statistical significance was calculated using two-way ANOVA followed by Sidak’s multiple comparisons test.

## Description

Injury, infection, oxidative or genotoxic stress, heat shock, or starvation disrupts organismal homeostasis and induces cellular stress pathways, which function to protect cells and promote recovery (Abramowicz & Pietrowska, 2019). AMPK is a heterotrimeric protein complex expressed in all eukaryotic cells composed of catalytic α and regulatory β and γ subunits (D G Hardie & Hawley, 2001; D Grahame Hardie, 2007). Functionally, AMPK acts as a master regulator of energy metabolism, responding to reduced energetics (i.e. AMP:ATP ratio). In mammals, phosphorylation of the α subunit at threonine 172 (T172) catalyzes AMPK activation (Willows et al., 2017). This site is conserved to threonine 243 (T243) of the AMPK ortholog AAK-2 of *C. elegans* (H. Lee, Cho, et al., 2008). The mammalian T172 and nematode T243 are phosphorylated by liver kinase β1 (LKB1) and abnormal embryonic partitioning of cytoplasm-4 (PAR-4), respectively (Hawley et al., 1996; H. Lee, Cho, et al., 2008). During favorable conditions, AMPK activity is kept at a basal level through regulation mechanisms that are largely unknown. Some evidence suggests that AMPK is inhibited by protein kinase A (PKA) (Djouder et al., 2010; Ferretti et al., 2016). In mammals, PKA phosphorylates AMPK at serine 173 (S173), which may sterically hinder phosphorylation at T172 (Djouder et al., 2010; Ferretti et al., 2016). This serine residue is conserved to position 244 (S244) of AAK-2, thus it may also serve a regulatory role in *C. elegans* , but this needs further investigation.

Using CRISPR we replaced T243 with an alanine *aak-2* (T243A) and S244 with a glycine *aak-2* (S244G). A widely used antibody for phosphorylated AMPK detects AAK-2 phosphorylation in wild-type animals at residue T243, however, it does not produce a band when used to analyze protein isolated from either mutant ( **Fig.1A** ). It is unclear if this is an accurate reflection of AMPK phosphorylation or if the antibody simply does not bind to the altered amino acid sequence. Thus, whole-animal western blot analyses are not possible when studying AMPK activation in the context of these mutations.

Whole-organism western blotting has provided valuable insight into the dynamics of AMPK activation (Jeong et al., 2018; Lee et al., 2008; Zhang et al., 2019). However, due to the limitations of using an antibody with our mutants, and the general challenges associated with western blotting, we aimed to use a genetically-coded AMPK biosensor, called AMPKAR-EV (Tsou et al., 2011; Kongaya et al., 2017). AMPKAR-EV, which has been expressed in cell culture and mice, uses fluorescence resonance energy transfer (FRET) to report AMPK activity *.* AMPKAR-EV is composed of the yellow and cyan fluorescent proteins (YFP and CFP), an AMPK substrate region, and a forkhead-associated 1 (FHA1) domain. Phosphorylation of the AMPK substrate region by active AMPK results in a conformation change caused by the binding of the AMPK substrate to the FHA1 domain. This reduces the distance between the donor (CFP) and acceptor molecules (YFP) and increases energy transfer ( **Fig.1B** ) *.* AMPKAR-EV includes a flexible EV linker (EV = extension for enhanced visualization by evading extraFRET) as a backbone that increases the dynamic sensitivity of the biosensor (Komatsu et al., 2011; Kongaya et al., 2017).

For this study, we expressed *C. elegans* codon adapted (Redemann et al., 2011) ceAMPKAR-EV in three tissues: intestine ( *nhx-2p* ::ceAMPKAR-EV), body wall muscle ( *myo-3p* ::ceAMPKAR-EV), and neurons ( *rab-3p* ::ceAMPKAR-EV) ( **Fig.1C** ). To initially test the functionality of the biosensor, we crossed the *nhx-2p* ::ceAMPKAR-EV strain with *unc-13* ( *e1091* ) animals, which are genetically immobilized (Richmond et al., 1999), for imaging purposes, and exposed them to a drug solution of paraquat and metformin, known AMPK activators (Meng et al, 2015; Wang et al., 2014). FRET ratios increased within minutes of drug exposure ( **Fig.1D** ) but did not change in control animals, suggesting the ceAMPKAR-EV biosensor can report AMPK activity *in vivo* .

To test this further, we crossed *nhx-2p::* ceAMPKAR-EV animals with both *aak-2* (T243A) and *aak-2* (S244G) mutant animals. In these experiments, we pre-treated animals for 10-minutes with paraquat and metformin solution and immobilized them with a solution of sodium azide, while in the presence of paraquat and metformin. Following treatment, wild-type animals displayed a significant increase in normalized FRET ratios over time, compared to untreated controls ( **Fig.1E** ), while *aak-2* (T243A) and *aak-2* (S244G) showed no change in normalized ratios between the treatment and control groups ( **Fig.1F** ). These data suggest that the T243 and S244 residues are required for the dynamics of AMPKAR-EV activation, but their exact role needs further investigation. Furthermore, considering *C. elegans* encodes two AMPK catalytic subunits, *aak-1* and *aak-2* (H. Lee, Cho, et al., 2008), it is possible that both subunits may be involved in AMPKAR-EV activation.

Next, AMPK activation was observed in a tail interneuron (DVA) and a ventral body-wall muscle, using *rab-3p* ::ceAMPKAR-EV and *myo-3p* ::ceAMPKAR-EV transgenic strains, respectively. The *rab-3p* ::ceAMPKAR-EV strain exposed to paraquat and metformin (with a 10-minute pre-exposure and immobilized with sodium azide) exhibited significantly higher normalized FRET ratios in the DVA ( **Fig.1G** ), while the *myo-3p* ::ceAMPKAR-EV strain showed no difference compared to controls ( **Fig.1H** ). These data suggest heterogeneity of AMPK activation exists between different tissues in *C. elegans.*

## Methods

Strains and worm handling

Animals were cultivated on NGM agar media and fed the *E. coli* strain DA837 (Avery & You, 2012). The worm strains used in this study were as follows: SJU333 = *stjEx216* [ *nhx-2p* ::ceAMPKAR-EV; *myo-2p* :: *mCherry* ], SJU335 = *aak-2* ( *stj20* )X; *stjEx216* – (S244G), SJU337 = *stjEx220* [ *myo-3p* ::ceAMPKAR-EV; *myo-2p* :: *mCherry* ], SJU345 = *aak-2* ( *stj17* )X; *stjEx216* – (T243A), SJU379 = *unc-13* ( *e1091* )I; *stjEx216* [ *nhx-2p* ::ceAMPKAR-EV; *myo-2p* :: *mCherry* ], SJU400 = *stjEx254* [ *rab-3p* ::ceAMPKAR-EV; *myo-2p* :: *mCherry* ].

ceAMPKAR-EV, cloning, and molecular biology

*C. elegans* codon adapted AMPKAR-EV followed by the *unc-54* 3’UTR was synthesized and cloned into the pUC57 vector (GeneScript©) (Redemann et al., 2011). The promoters for the genes *nhx-2* (intestine), *myo-3* (muscle), and *rab-3* (neurons) were amplified from genomic DNA by PCR, with engineered ApaI and NheI restriction sites at the 5’ and 3’ end, respectively. The primer sequences used were as follows: *nhx-2p* : ATATGGGCCCCAATGGCTGAAGGACCGAAAC and ATATGCTAGCGATTTAATCACTGAAAATTATTTC; *myo-3p* : ATATGGGCCCGTGATTATAGTCTCTGTTTTCGTTAAT and ATATGCTAGCATTTCTAGATGGATCTAGTGGTC; *rab-3p* : ATATGGGCCCTTAACTAGTCAATCTTTCTCTCTTTTTC and ATATGCTAGCAAATCAAATTTTTAAATGCATTT. Each promoter was cloned 5’ of the ceAMPKAR-EV sequence by restriction digest and ligation. Transgenesis was performed by microinjection, as described (Mello and Fire, 1995), all plasmids/constructs were injected at 25 ng/µl with 5 ng/µl pCFJ90( *myo-2p* :: *mCherry* ) and 120 ng/µl 1 kb ladder (New England Biolabs ©).

Construction of mutants using CRISPR

The strains SJU335 and SJU345 were constructed by CRISPR/Cas9 gene editing, using a published protocol (Arribere et al., 2014). A mixture of guide RNA (gRNA) (GACTTCTTACGCACCAGCTGCGG) duplexed with Alt-R® CRISPR-Cas9 tracrRNA (IDT©), Alt-R® S.p. Cas9 Nuclease V3 (IDT) and oligonucleotide repair templates (T243A: TGGACTTTCAAATATTATGACGGATGGTGACTTCTTACGCGCTAGCTGCGGATCGCCAAATTATGCTGCCCCTGAGGTTA; or, S244G: TTTGGACTTTCAAATATTATGACGGATGGTGACTTCTTACGCACCGGTTGCGGATCGCCAAATTATGCTGCCCCTGAGGTTATT) were injected into day-1 adult wild-type animals. Simultaneously, an edit of the *dpy-10* gene was made to allow for screening, as described (Arribere et al., 2014). Dpy or Rol progeny of the injected animals were transferred to individual plates, maintained to the next generation, and screened by PCR and restriction digest. The *aak-2* (T243A) and *aak-2* (S244G) mutations introduced NheI and AgeI sites, respectively. Both mutations were confirmed by sequencing and subsequently crossed to SJU333.

Microscopy, FRET imaging and analysis

All experiments were performed with day-1 adult hermaphrodites. Paraquat and metformin solutions were prepared the day of imaging: 40mM paraquat, 40mM metformin suspended in *E. coli* DA837 culture. The SJU379 strain was placed in a 4.5μL drop of the drug solution supplemented with 2μl of 50mM sodium azide. All other animals were pre-treated for 10 minutes in a 4.5μL drop of drug solution and immobilized by supplementing the solution with 2 μL of 50mM sodium azide. Control animals were treated the same as above but only exposed to *E. coli* DA837 culture without drugs. Animals were mounted on NGM agarose pads and imaged every 2 minutes on an Olympus IX81 inverted fluorescent microscope equipped with a Photometrics DualView image splitter for simultaneous CFP and YFP imaging. Images were acquired with an aqua longpass excitation filter ET436/30M (Chroma 19001) and a DV2-chameleons-cube kit (Chroma 505lpxr), containing a dichroic mirror and two emission filters (ET480/30m, ET535/30m). Split CFP/YFP images were analyzed using Cellsens software. CFP and YFP intensity were obtained for a defined ROI, and background was subtracted. For each image, the YFP intensity was divided by CFP intensity to produce the raw FRET ratio (YFP:SECFP). FRET ratios at each time point were normalized to that of the first image (t=0 or t=10 for pre-treated animals). Each experimental group was compared to a control of the same strain and statistical significance was calculated using two-way ANOVA followed by Sidak’s multiple comparisons test.

The images displayed in **Fig.1C** were obtained using an Olympus BX63 wide-field fluorescent microscope under the FITC channel.

Western blotting

First-day adults were collected in extraction buffer containing Laemmli buffer, 2-mercaptoethanol, and protease/phosphatase inhibitors (Halt™ Protease Inhibitor Cocktail - Catalog #78430; PhosSTOP™ - Product # 4906845001). Samples were heated and run on a 4–20% Criterion™ TGX™ Precast Midi Protein Gel (Product #5671094), transferred to a nitrocellulose membrane (GE Healthcare Amersham™ Protran™ NC Nitrocellulose Membranes - Catalog #45-004-007), and blotted using phospho-AMPKα (Thr172) antibodies from Cell Signaling Technology© (mAb #2535). Membranes were stripped and blotted again with 12G10 α-tubulin (DSHB), as a loading control.
